# Virulence Markers and Phylogenetic Analysis of *Escherichia coli* Strains with Hybrid EAEC/UPEC Genotypes Recovered from Sporadic Cases of Extraintestinal Infections

**DOI:** 10.3389/fmicb.2017.00146

**Published:** 2017-02-03

**Authors:** Flaviane B. M. Lara, Danielly R. Nery, Pâmela M. de Oliveira, Mayana L. Araujo, Fabiana R. Q. Carvalho, Lorena C. F. Messias-Silva, Leonardo B. Ferreira, Celio Faria-Junior, Alex L. Pereira

**Affiliations:** ^1^Graduate Program in Microbial Biology, Biology Institute, University of BrasíliaBrasília, Brazil; ^2^Campus of Ceilândia, University of BrasíliaBrasília, Brazil; ^3^Ceilândia Regional Hospital, Secretary of State for HealthBrasília, Brazil; ^4^Central Laboratory for Public Health, Secretary of State for HealthBrasília, Brazil

**Keywords:** enteroaggregative *Escherichia coli*, uropathogenic *Escherichia coli*, hybrid strain, genotyping, phylogenetic group, multilocus sequence typing

## Abstract

Virulence genes from different *E. coli* pathotypes are blended in hybrid strains. *E. coli* strains with hybrid enteroaggregative/uropathogenic (EAEC/UPEC) genotypes have sporadically emerged causing outbreaks of extraintestinal infections, however their association with routine infections is yet underappreciated. We assessed 258 isolates of *E. coli* recovered from 86 consecutive cases of extraintestinal infections seeking EAEC and hybrid genotype (EAEC/UPEC) strains. Extensive virulence genotyping was carried out to detect 21 virulence genes, including molecular predictors of EAEC and UPEC strains. Phylogenetic groups and sequence types (STs) were identified, as well as it was performed phylogenetic analyses in order to evaluate whether hybrid EAEC/UPEC strains belonged to intestinal or extraintestinal lineages of *E. coli*. Adhesion assays were performed to evaluate the biofilm formation by hybrid strains in human urine and cell culture medium (DMEM). Molecular predictors of UPEC were detected in more than 70% of the strains (*chuA* in 85% and *fyuA* in 78%). Otherwise, molecular predictors of EAEC (*aatA* and *aggR*) were detected in only 3.4% (9/258) of the strains and always along with the UPEC predictor *fyuA*. Additionally, the pyelonephritis-associated pilus (*pap*) gene was also detected in all of the hybrid EAEC/UPEC strains. EAEC/UPEC strains were recovered from two cases of community-onset urinary tract infections (UTI) and from a case of bacteremia. Analyses revealed that hybrid EAEC/UPEC strains were phylogenetically positioned in two different clades. Two representative strains, each recovered from UTI and bacteremia, were positioned into a characteristic UPEC clade marked by strains belonging to phylogenetic group D and ST3 (Warwick ST 69). Another hybrid EAEC/UPEC strain was classified as phylogroup A-ST478 and positioned in a commensal clade. Hybrid EAEC/UPEC strains formed biofilms at modest, but perceptible levels either in DMEM or in urine samples. We showed that different lineages of *E. coli*, at least phylogenetic group A and D, can acquire and gather EAEC and UPEC virulence genes promoting the emergence of hybrid EAEC/UPEC strains.

## Introduction

*Escherichia coli* colonizes the human intestine few hours after birth establishing a mutually beneficial relationship with its hosts. While they are restricted to the outer layer of intestinal mucus, these commensal *E. coli* strains rarely cause infections. However, some highly adapted *E. coli* lineages have evolved acquiring a broad range of virulence genes (VGs) that allows *E. coli* strains to adapt to, colonize and invade several anatomic sites (Kaper et al., 2004).

Pathogenic and commensal strains of *E. coli* are differently sorted into four major phylogenetic groups (phylogroups) named as A, B1, B2, and D (Doumith et al., 2012). Epidemiological studies have shown that extraintestinal pathogenic *E. coli* (ExPEC) strains are frequently classified as phylogroup B2 or D, while commensal strains are frequently sorted into phylogroup A or B1. Nevertheless, mechanisms of horizontal genetic transfer allows the exchange of VGs among phylogroups, which may promote the sporadic emergence of highly virulent strains belonging to commensal phylogroups A or B1. Additionally, each *E. coli* phylogroup may enclose heterogeneous groups of strains and different clonal populations, fact which imposes a more complex scenario in an attempt to establish epidemiological links between *E. coli* phylogroups and human infections (Dias et al., 2009). Thereafter, characterizing clonal structure within each phylogroup seems to be important toward recognizing subsets of clonal groups associated with distinct clinical features. The development of multilocus sequence typing (MLST) methods and the subsequent definition of sequence types (STs) pave the way to the recognition of highly virulent ExPEC clones with worldwide dispersion, such as the clones B2-ST131 and D-ST69 (Blanco et al., 2011; Nicolas-Chanoine et al., 2014; Petty et al., 2014).

In *E. coli*, successful VG combinations have persisted among strains leading to the recognition of *E. coli* pathotypes. Infections with *E. coli* pathotypes can result in three common clinical conditions: diarrhea, urinary tract infection (UTI) and sepsis/meningitis. Diarrheagenic *E. coli* (DEC) strains are sorted into six well-defined pathotypes, each displaying distinctive VG arrangements: enteropathogenic *E. coli* (EPEC), enterohemorrhagic *E. coli* (EHEC), enterotoxigenic *E. coli* (ETEC), enteroinvasive *E. coli* (EIEC), diffusely adherent *E. coli* (DAEC), and enteroaggregative *E. coli* (EAEC) (Kaper et al., 2004).

On a molecular basis, an EAEC strain is classified as the isolate carrying the virulence plasmid pAA, which is marked by the presence of the predictor genes *aatA* (previously named as CVD432 probe) and *aggR* (the master virulence regulator in EAEC). Additionally, the pAA may harbor other EAEC-specific VGs, including alleles of aggregative adherence fimbriae (*aggA, aafA agg-3A, hdaA, agg-5A*), dispersin gene (*aap*), and the plasmid-encoded toxin gene (*pet*). EAEC prototype strain 042 has an archetypal pAA plasmid displaying a complete set of virulence genes: *aatA, aggR, aafA, aap*, and *pet*. However, epidemiological studies have shown that wild type strains maintain pAA plasmids with a virulence load quite different from the one from archetypal EAEC plasmids. As an example, wildtype EAEC strains collected in epidemiological approaches may show a low frequency for the detection of aggregative fimbriae genes and *pet* (Pereira et al., 2007). Given their genetic plasticity, a minimum set of predictor genes (the occurrence of *aatA* along with *aggR*) was proposed to define typical EAEC strains (Kaper et al., 2004).

Molecular analyses of *E. coli* strains isolated from UTIs, bacteremia and neonatal meningitis lead to the recognition that these strains are phylogenetically distinct from commensal strains. Contrary to commensal strains, those pathogenic strains frequently belong to the phylogoup B2 or D (Russo and Johnson, 2000). Thereafter, the term extraintestinal pathogenic (ExPEC) was coined to classify strains recovered from extraintestinal infections but that appear to be incapable of causing diarrhea (Russo and Johnson, 2000). Differently from DEC infections, ExPEC infections rely on the bacterial translocation from intestinal lumen to an extraintestinal site, which frequently is the urinary tract.

*E. coli* strains recovered from UTI (uropathogenic *E. coli*—UPEC) are the most common cause of bacterial infections in humans, mainly among women, and account for around 100 million cases a year (Foxman, 2010). UPEC strains are a heterogeneous category of *E. coli* displaying a considerable number of well-recognized VGs (**Table 2**) which can be combined into different genotypes. Despite that diversity, a set of four VGs has been reported as a predictor of UPEC strains. Any two of the genes *chuA* (heme receptor), *yfcV* (YfC fimbria) or *vat* (vacuolating autotransporter protein), when detected along with the gene *fyuA* (yersiniabactin siderophore receptor), can be used to differentiate UPEC strains from commensal and DEC strains (Spurbeck et al., 2012). Besides those VGs, the genes encoding fimbria P (*pap*, standing for pyelonephritis-associated pilus) and fimbria F1C (*focA*) play a pivotal role in ascending UTIs since they promote the colonization of proximal and distal tubular cells from human kidney (Korhonen et al., 1986; Marre et al., 1990).

Bacterial biofilms play an important role in medicine and is a serious issue mainly in urology. Bacteria that adhere to uroepithelial cells and form biofilms are more prone to cause pyelonephritis and even chronic or recurrent infections (Soto, 2014). Several studies reported that most of isolates collected from patients with recurrent infections were biofilm producers *in vitro*. Aside UPEC strains, the expression of biofilms has been considered a consensual virulence factor among EAEC isolates. In EAEC strains, biofilm formation is a complex event that may involve multiples adhesins and factors not devoted to adhesion (Pereira et al., 2010).

The remarkable genome plasticity displayed by *E. coli* strains has allowed the emergence of virulent strains displaying unusual arrangement of VGs, including arrays of VGs from different pathotypes detected in a single isolate (Gomes et al., 2016). Considering molecular markers from all diarrheagenic *E. coli* (DEC) pathotypes, EAEC virulence genes are among the most frequent markers of DEC strains reported in ExPEC strains isolated from sporadic cases of extraintestinal infections and outbreaks (Abe et al., 2008; Aurass et al., 2011; Olesen et al., 2012; Prager et al., 2014; Toval et al., 2014; Ang et al., 2016). The term “heteropathogenic *E. coli*” is now adopted to designate pathogenic *E. coli* strains that maintain phenotypic and genetic determinants from different *E. coli* pathotypes (Bielaszewska et al., 2014; Toval et al., 2014; Ang et al., 2016). This intriguing epidemiological scenario has shown how artificial and limited is the current genetic classification of strains into classical pathotypes of *E. coli* (Robins-Browne et al., 2016).

This study aimed to search for hybrid EAEC/UPEC strains recovered from sporadic extraintestinal infections in order to identify their genotypes and phylogenetic position among UPEC strains.

## Materials and methods

### Ethics statement

This study was approved by the FEPECS research ethics committee, which is linked to the Secretary of State for Health (Brasília-DF), under the registry number 782.067.

### Samples and strains

During a period of 5 months, microbiological samples were recovered from patients with *E. coli*-associated extraintestinal infections attended to in a tertiary hospital in Brasília-DF, Brazil. Two hundred fifty eight isolates of *E. coli* (3 isolates for each sample) were isolated from 86 consecutive cases of extraintestinal infections including urinary tract infection (79 cases); bacteremia (1 case); pneumonia (1 case); surgical site infection (2 cases); peritoneal cavity infection (1 case); and mucosa infections (2 cases) (Table 1).

**Table 1:** **Characteristics of the 86 patients enrolled in this study**.

			**Patient type**		
			**Outpatient (%)**	**Inpatient (%)**	**Not specified**
			64 (74.4)	21 (24.4)	1 (1.2)
**Gender**	***F***		47	11	–
	***M***		17	10	1
**Age**		Mean ± SD	31.6 ± 28.4	48.7 ± 28.1	3
		Median	27	56	3
		Minimum	0	0	3
		Maximum	91	88	3
**Infections**		Urinary tract infection	62 (96.8)	16 (76.1)	
		Bacteremia	0	1 (4.7)	
		Pneumonia	0	1 (4.7)	
		Surgical site infection	0	2 (8.2)	
		Peritoneal cavity infection	0	1 (4.1)	
		Mucosa infections	2 (3.1)	0	

### Genotyping

The detection of virulence genes was carried out by the polymerase chain reaction (PCR). Supernatants derived from bacterial suspension treated by boiling were used as the source of DNA template. All strains were tested for the presence of 8 virulence markers of UPEC (*fyuA, yfcV, chuA, vat, focA, pap, sfa, cnf*), fimbriae curli (*csgA* gene), antigen 43 adhesin (*ag43*), plasmid-encoded type IV pilus (*pilS*), and the EAEC mucinase gene (*pic*) (Table 2). In order to detect EAEC strains, three EAEC molecular predictors were also tested in all strains: *aatA* (formerly pCVD432 probe) *aggR* (EAEC virulon regulator gene) and *aapA* (dispersin gene) (Table 2). The presence of five alleles of aggregative adherence fimbriae (AAF-I to AAF-V) were tested in strains positive for *aatA* or *aggR*. The prototype strains 042 (positive for *aatA, aggR, aapA, aafA, astA, pet*, and *pic*) and 17-2 (positive for *aatA, aggR, aapA, aggA* and *astA*) were used as positive controls for the detection of EAEC markers. The Pasteur Institute-referenced strains FVL2 (positive for *fyuA, yfcV, chuA, vat, sfa, pap, cnf*) and FV34 (positive for *fyuA, yfcV, chuA, vat, pap, sfa*) were used as positive controls for UPEC markers. Primers designed in this study (Table 2) were constructed on based of the following GenBank sequences: FN554767.1 from EAEC 042 (primers *aapA*); EU637023.1 from *E. coli* C1010-00 (primers *hdaB-A*); KP202151.1 from *E. coli* plasmid pAA Agg5A (primers *agg-5A*); HG941718.1 from *E. coli* EC958 (primers *fyuA*); CP000243.1 from *E. coli* UTI89 (primers *yfcV*); AF280396.1 from *E. coli* hemin receptor ChuA (primers *chuA*); KR094957.1 from *E. coli* PAB72 (primers *vat*); AF298200.1 from *E. coli* AD110 (primers *focA*); AF483829.1 from *E. coli* 5383-2 (primers *cnf*); L04979.1 from *E. coli* curli gene (primers *csgA*); and, AF233272.1 from *E. coli* antigen 43 precursor gene (primers *ag43*).

**Table 2:** **List of primers used in the virulence genotyping**.

**Virulence marker**	**Locus description**	**Primers**	**Frag. (bp)**	**Tm (°C)**	**References**
**EAEC VIRULENCE GENES**					
*aatA*	Dispersin transporter (earlier pCVD432)	CTGGCGAAAGACTGTATCAT	630	60	Pereira et al., 2007
		CCATGTATAGAAATCCGCTGTT			
*aggR*	Transcription acti*vat*or	CTAATTGTACAATCGATGTA	324	50	Pereira et al., 2010
		CTGAAGTAATTCTTGAAT			
*aapA*	Dispersin	CTTTTCTGGCATCTTGGGT	328	56	This study
		TTATTTAACCCATTCGGTTAGAGC			
*aggA*	Aggregative adherence fimbria (AAF-I)	GCTAACGCTGCGTTAGAAAGACC	421	60	Pereira et al., 2007
		GGAGTATCATTCTATATTCGCC			
*aafA*	AAF-II	GACAACCGCAACGCTGCGCTG	233	50	Pereira et al., 2007
		GATAGCCGGTGTAATTGAGCC			
*agg-3A*	AAF-III	GTATCATTGCGAGTCTGGTATTCAG	462	56	Bernier et al., 2002
		GGGCTGTTATAGAGTAACTTCCAG		58	
*hdaB-A*	AAF-IV	CTGTAGGACGTAGGTAATGAAACTG	993		This study
		AAACTCCAGGCGTTAACGTCTG			
*agg-5A*	AAF-V	AGCAGCAACAGCAAATCC	342	56	This study
		CCGTAACCACTTCCTCGT			
*pet*	Plasmid-encoded toxin	CCGCAAATGGAGCTGCAAC	1133	60	Pereira et al., 2007
		CGAGTTTTCCGCCGTTTTC			
*pic*	Mucinase	TTCAGCGGAAAGACGAA	500	60	Pereira et al., 2007
		TCTGCGCATTCATACCA			
**UPEC VIRULENCE GENES**					
*fyuA*	Yersiniabactin siderophore receptor	TGAGTGGGAAATACACCACC	715	54	This study
		TTACCCGCATTGCTTAATGTC			
*yfcV*	YfC fimbria	ATCCGTGTTGGCTGGC	280	54	This study
		GGTCATGGGCGCAGTT			
*chuA*	Heme receptor	TAACTGTCATAGCGGGTTCC	439	55	This study
		AGTCTCTGAGCGGTTTAGTG			
*vat*	Vacuolating autotransporter toxin	CAGAACATTTGCTCCCTTGT	1102	53	This study
		ACACGTTCAGGATTCAGT			
*papC*	Pyelonephritis-associated pilus	GACGGCTGTACTGCAGGGTGTGGCG	328	60	Daigle et al., 1994
		ATATCCTTTCTGCAGGGATGCAATA			
*focA*	Fimbria F1C (*focA-sfaD*)	GAAAGTAGATGGAGCTAAAAGCAAT	496	54	This study
		CATGACATGCCAGTGGTTTC			
*sfa*	Fimbria S	CTCCGGAGAACTGGGTGCATCTTAC	407	60	Daigle et al., 1994
		CGGAGGAGTAATTACAAACCTGGCA			
*cnf*	Cytotoxic necrotizing factor	GTGAAGCTCAACGAGACTAT	826	53	This study
		TCAGTAGCTCCTCTCATCAA			
**GENERAL *E. COLI* ADHESINS**					
*csgA*	Curli fimbria	CTCTGACTTGACTATTACC	199	50	This study
		AGATGCAGTCTGGTCAAC			
*ag43*	Self-recognizing protein (adhesin)	CGATCGATAAGCTAATAATAACC	552	55	This study
		GAAGACCACCACTGGTGACA			
*pilS*	Plasmid-encoded type IV pili	ATGAGCGTCATAACCTGTTC	532	58	Dudley et al., 2006

### Phylogrouping, MLST, and phylogenetic tree

Definition of major *E. coli* phylogroups (A, B1, B2, and D) was performed by PCR as described by Doumith et al. (2012). Multilocus sequence typing (MLST) was performed in accordance with the Institute Pasteur scheme using eight housekeeping genes (*dinB, icdA, pabB, polB, putP, trpA, trpB*, and *uidA*) (Jaureguy et al., 2008). Procedures, primers and the sequence type (ST) assignment were carried out as described at Pasteur's webpage (http://bigsdb.pasteur.fr/ecoli/ecoli.html). BigDye Terminator v3.1 Cycle Sequencing Kit (Applied Biosystems™) was employed in sequencing reactions and amplicons were analyzed with ABI-Prism 3500 Genetic Analyzer (Applied Biosystems™). Clonal complexes (CC) were defined as groups of two or more independent isolates that shared identical alleles at six *loci*. The correspondence between STs assignment by Pasteur's scheme and Achtman's scheme was based on results published in previous studies (Jaureguy et al., 2008; Clermont et al., 2015). In order to display the phylogenetic relationships among strains, MLST sequences were concatenated into a 2901-base-long super-gene (*dinB*-*icdA*-*pabB*-*polB*-*putP*-*trpA*) (Gadagkar et al., 2005). The dendrogram was constructed in MEGA6 applying the Maximum Likelihood method based on the Tamura-Nei model (Tamura et al., 2013). Initial trees for the heuristic search were obtained by applying the Neighbor-Joining method to a matrix of pairwise distances estimated using the Maximum Composite Likelihood (MCL) approach. In order to test the accuracy of the phylogeny was applied the Bootstrap method with 1500 replications.

### Samples of human urine

Healthy women with no history of consumption of antibiotics or anti-inflammatoreis within the last 15 days and with no clinical urinary symptoms were selected to donate urine samples. Donators were instructed, informed about the absence of health risk associated with the urine collection, and signed an informed consent term allowing the urine collection. Morning samples of urine (volume of 50 mL) were collected by spontaneous urination, centrifuged (3000 g/3 min), sterilized by ultrafiltration (0.22 μm) and preserved at −20°. Samples of sterile urine were pooled (*n* = 3), supplemented with 0.5% of casamino acid and used as culture medium in biofilm assays.

### Biofilm assays

In order to test the biofilm formation, 96-well flat-bottom polystyrene plates were used as described by Wakimoto et al. (2004). Seventy seven strains (one strain per case including 3 hybrid EAEC/UPEC strains) were assayed for biofilm formation (Supplementary Table 1). Briefly, 200 μL of Dulbecco's Modified Eagle Medium (DMEM) or sterile samples of pooled human urine were set into each plate well and inoculated with 5 μL of overnight bacterial culture. The plates were incubated overnight at 37°C without shaking. Afterwards, planktonic culture were discarded and formed biofilms were stained with crystal violet (CV) dye (15 min), washed once with 200 μL of phosphate-buffered saline and air-dried for 3 h. The absorbance (OD at 630 nm) reached by CV adsorbed on the well bottom was determined, and afterwards the bacterium-bound dye was released by the addition of ethanol (200 μL/well). The mean of the absorbances was used as a measure of the formed biofilms. Data were displayed as means obtained from three independent assays.

### Statistical analysis

Statistical analysis were performed on the software IBM® SPSS® Statistics (version 20). Results with *p* ≤ 0.05 were considered to be statistically significant.

## Results

### Phylogroups and virulence genes among ExPEC strains

Cases of extraintestinal infections were predominantly associated with phylogroup B2 (37/86–43%) and D (33/86–38%), followed by the phylogroups B1 (6/86–6.9%) and A (5/86–5.8%) (Data not shown). In three cases of UTIs, different phylogroups were simultaneously recovered: A/B2; B1/D and B2/D.

The molecular predictors of UPEC *chuA* (85%) and *fyuA* (78%) were predominantly detected among ExPEC strains. Additionally, the genes for UPEC-specific fimbriae *pap* and *yfcV* were detected in 47 and 41% of the strains, respectively (Table 3). UPEC VGs displayed an uneven distribution among phylogroups. The genes *fyuA, pap* e *yfcV* were more frequently detected in the extraintestinal phylogroups (B2 and D) than in the intestinal groups (A and B1) (*p* < 0.001—Fisher's exact test) (Table 3). Moreover, the genes *sfa, cnf* and *pic* were exclusively detected in the phylogroup B2.

**Table 3:** **Distribution of virulence genes among phylogenetic groups of ***E. coli*****.

**Phylogenetic groups**							
	**General positivity**		**Intestinal**		**Extraintestinal**		**Sig. (Kruskal-Wallis)**
	***N*** = **258**	NT^*^N = **42**	**A** ***N*** = **16**	**B1** ***N*** = **16**	**B2** ***N*** = **96**	**D** ***N*** = **88**	
	**Count (%)**	**Count (%)**	**Count (%)**	**Count (%)**	**Count (%)**	**Count (%)**	
**GENES**							
*chuA* ^a^	219 (85)	35 (83)	0	0	96 (100)	88 (100)	–^a^
*csgA*	213 (83)	27 (64)	10 (63)	10 (63)	84 (88)	82 (93)	0.000
*fyuA* ^b^	200 (78)	31 (74)	4 (25)	0	93 (97)	72 (82)	0.000
*pap* ^b^	120 (47)	22 (52)	3 (19)	2 (13)	46 (48)	47 (53)	0.003
*yfcV* ^b^	106 (41)	14 (33)	0	0	73 (76)	19 (22)	0.000
*vat*	102 (40)	12 (29)	10 (63)	3 (19)	62 (65)	15 (17)	0.000
*cnf* ^c^	60 (23)	8 (19)	0	0	52 (54)	0	0.000
*focA*	45 (17)	6 (14)	12 (75)	6 (38)	10 (10)	11 (13)	0.000
*sfa* ^c^	39 (15)	5 (12)	0	0	34 (35)	0	0.000
*ag43*	21 (8)	1 (2)	3 (19)	0	9 (9)	8 (9)	0.343
*aapA*	12 (5)	3 (7)	3 (19)	0	0	6 (7)	0.002
*pic* ^c^	11 (4)	0	0	0	11 (11)	0	0.002
*aatA*	9 (3)	0	3 (19)	0	0	6 (7)	0.001
*aggR*	9 (3)	0	3 (19)	0	0	6 (7)	0.001
*pilS*	9 (3)	2 (5)	0	0	3 (3)	4 (5)	0.672
*aggA*	3 (1)	0	3 (19)	0	0	0	0.000
*pet*	0	0	0	0	0	0	1
*aafA*	0	0	0	0	0	0	1
*agg3A*	0	0	0	0	0	0	1
*hdaA*	0	0	0	0	0	0	1

* *Not tested for phylogenetic groups*.

“a” *– the presence of gene chuA is mandatory for the classification into phylogroups B2 or D*.

“b” *indicates virulence genes more statistically detected in extraintestinal strains (B2+D) in comparison with intestinal ones (A+B1) (p < 0.001)*.

“c” *indicates virulence genes exclusively detected in the phylogroup B2*.

The molecular predictors of EAEC *aatA* and *aggR* were co-detected in 3.4% (9/258) of the ExPEC strains recovered from 3 out of 86 studied cases (3.4%). Besides *aatA* and *aggR*, the UPEC genes *fyuA* and *pap* were always found forming the genotypes of these strains, which highlighted their hybrid nature: hybrid EAEC/UPEC strains (Table 4). Hybrid EAEC/UPEC strains were recovered from two cases of UTIs (cases 63 and 85) and from one case of bacteremia (case 17) (Table 4). Additionally, three strains were classified as phylogroup A (associated with the UTI case 63) and the six remaining strains were classified as phylogroup D (with each of the three strains associated with the UTI case 85 and the bacteremia case 17). Genes for the EAEC fimbria AAF-I (*aggA*) were also detected in the EAEC/UPEC strains isolated from the UTI case 63 (Table 4). Aside from these data, an interesting fact was that three strains recovered from an UTI case (case 55) tested positive for the EAEC dispersin gene (*aapA*), although they did not harbor any of the EAEC-predictor markers *aatA* or *aggR* (Table 4).

**Table 4:** **Cases of extraintestinal infections associated with strains harboring virulence genes of EAEC**.

						**EAEC markers**				**UPEC markers**							
**Case (Identification number)**	**In or Outpatient**	**Gender**	**Age (year)**	N of strain^*^	**Pathotype**	***aatA***	***aggR***	***aapA***	***aggA***	***fyuA***	***chuA***	***vat***	***pap***	***focA***	***csgA***	***ag43***	**Phylogroup**
Bacteremia (17)	In	F	62	3	EAEC/UPEC												**D**
UTI (63)	Out	M	74	3	EAEC/UPEC												**A**
UTI (85)	Out	F	1	3	EAEC/UPEC												**D**
UTI (55)	Out	F	-	3	UPEC												**D**

* *All strains isolated from each case displayed the same genotype*.

### Allelic profile and sequence type (ST) in hybrid EAEC/UPEC strains

Three hybrid EAEC/UPEC strains (each of them isolated from the cases 17, 63, and 85) had their allelic profiles and STs determined and compared with 19 contemporaneous UPEC strains, each from a different case chosen randomly (Table 5). Additionally, the equivalent Warwick ST was assessed in accordance with a previous report (Clermont et al., 2015) (Table 5). Twelve allelic profiles were detected among the strains. The predominant allelic profile matched exactly with ST3 (Warwick ST69) (*n* = 8) and was followed by ST43 (*n* = 3) (Warwick ST 131). Five STs were of single occurrence (ST8, ST34, ST44, ST478, and ST479), and two STs were single-locus variants of ST43 and ST4 (Warwick ST73) (Table 5). Concerning the hybrid EAEC/UPEC strains, two strains (17.1 and 85.1) were classified as ST3, (Warwick ST69) while the strain 63.1 was addressed to the ST478, which had a single occurrence. These findings uncover the heterogeneity of our collection and show that a considerable subset of strains (*n* = 11) belong to genetic lineages recognized by the epidemic potential (ST3 and ST43), including two of the hybrid EAEC/UPEC strains.

**Table 5:** **Allelic profile of hybrid EAEC/UPEC and UPEC strains**.

**Strain**	**Pathotype**	**Sample**	**Allelic profile—Pasteur scheme**									**Equivalent Warwick^a^**
			***dinB***	***icdA***	***pabB***	***polB***	***putP***	***trpA***	***trpB***	***uidA***	**ST**	**ST**
**17.1**	**EAEC/UPEC**	Blood	3	8	5	11	8	3	5	3	3	69
**85.1**	**EAEC/UPEC**	Urine	3	8	5	11	8	3	5	3	3	69
9.1	UPEC	Urine	3	9	5	11	8	3	5	3	3	69
16.1	UPEC	Urine	3	8	5	11	8	3	5	3	3	69
19.1	UPEC	Urine	3	8	5	11	8	3	5	3	3	69
28.1	UPEC	Urine	3	8	5	11	8	3	5	3	3	69
38.1	UPEC	Urine	3	8	5	11	8	3	5	3	3	69
76.1	UPEC	Urine	3	8	5	11	8	3	9	ND^b^	3	69
12.1	UPEC	Urine	9	1	15	7	4	9	6	9	43	131
8.1	UPEC	Urine	9	1	15	7	4	9	145	9	SLV^d^43	131
21.1	UPEC	Urine	9	1	15	7	4	9	6	9	43	131
58.1	UPEC	Urine	9	1	15	7	4	9	6	9	43	131
81.1	UPEC	Urine	2	4	6	4	1	6	1	25	SLV^d^4	73
**63.1**	**EAEC/UPEC**	Urine	8	2	7	84	7	1	ND^b^	2	478	-
23.1	UPEC	Secretion	12	45	24	19	13	24	8	29	34	-
50.1	UPEC	Urine	23	9	8	12	8	11	7	13	8	-
73.1	UPEC	Urine	17	9	28	12	9	13	5	11	44	-
84.1	UPEC	Urine	9	37	4	146	78	8	2	30	479	-
6.1	UPEC	Urine	30	45	33	37	27	34	24	NA^c^	**-**	**-**
11.1	UPEC	Urine	30	45	33	37	27	34	24	NA^c^	**-**	**-**
57.1	UPEC	Urine	30	45	33	37	27	34	24	NA^c^	**-**	**-**
27.1	UPEC	Urine	30	45	24	37	27	34	24	NA^c^	**-**	**-**

a *Defined in accordance with Clermont et al. (2015)*.

b *Allele did not define*.

c *Allele did not amplify*.

d *SLV – Single-locus variant*.

### Phylogenetic positioning of hybrid EAEC/UPEC strains

**Figure 1:**
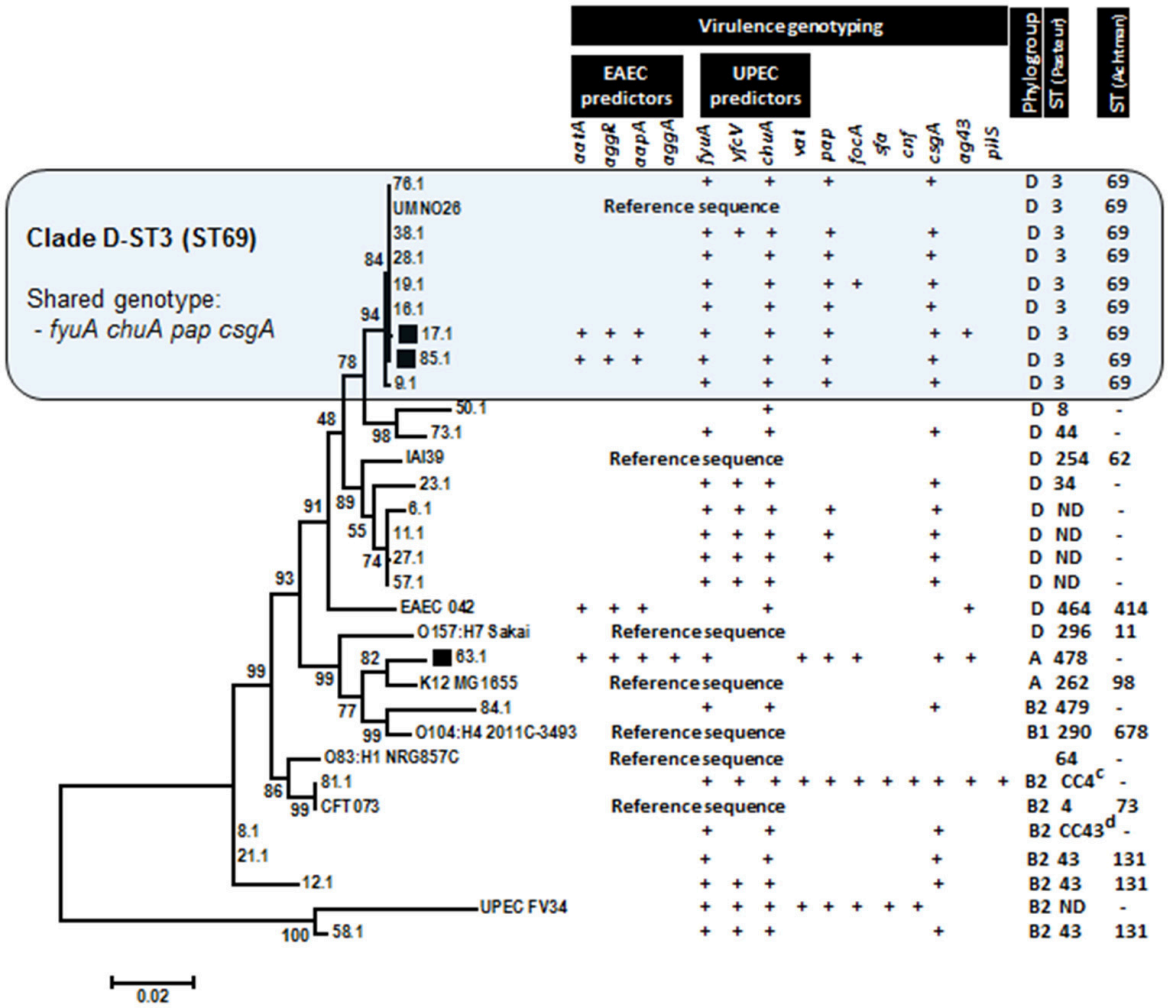
**Phylogenetic relationship among ***E. coli*** strains inferred with Maximum Likelihood method (1500 replication) by using 2901-base-long concatenated sequences ***dinB-icdA-pabB-polB-putP-trpA*****. Concatenated sequences derived from sequenced genomes of prototype strains were adopted to characterize the phylogenetic clades. Hybrid EAEC/UPEC strains (indicated by black squares) were positioned in two different clades, including a large one shared by typical UPEC strains (Clade D-ST3).

The phylogenetic relationship of hybrid EAEC/UPEC with typical UPEC strains, prototype EAEC 042, and genomic reference sequences were visualized in a dendrogram constructed by alignments of concatenated housekeeping gene sequences (*dinB-icdA-pabB-polB-putP-trpA*) (Figure 1). Genotypes, phylogroups and STs were also displayed in order to highlight shared features detected among strains. Two of out three tested EAEC/UPEC strains shared with UPEC strains a well-defined clade (bootstrap value equals 94) featured by gathering strains (*n* = 8) with positive results for molecular predictors of UPEC (*fyuA* and *chuA*) and UPEC-specific fimbria Pap as well (Figure 1). Additionally, strains in this clade were addressed as phylogroup D and ST3 (Warwick ST 69). Outside the clade D-ST3, the strains showed a reduced positivity for *pap* (40% - 6/15). The prototype EAEC strain 042 was positioned in a separate clade, although it also belongs to phylogroup D. A different phylogenetic position was displayed by the EAEC/UPEC strain 63.1, which was positioned in a different major branch sharing a clade with the reference sequence of commensal strain K12. Endorsing that phylogenetic positioning is the fact that both strains belong to phylogroup A (Figure 1).

### Biofilm formation by hybrid EAEC/UPEC stains

**Figure 2:**
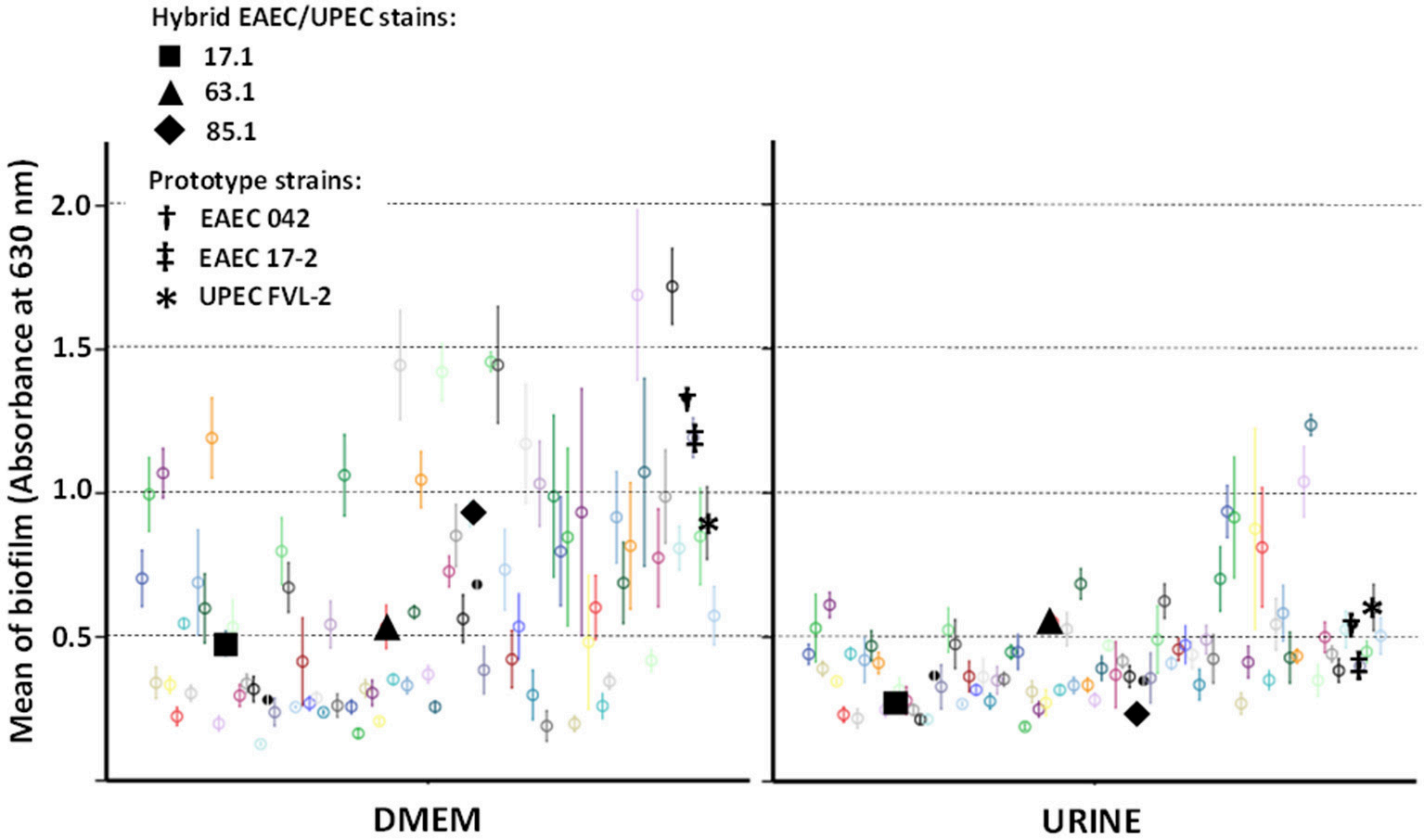
**Biofilm formed by ***E. coli*** strains in Dulbecco's Modified Eagle Medium (DMEM) and sterile samples of pooled human urine**. Hybrid EAEC/UPEC and prototype strains are highlighted with black solid and bolded symbols (respectively). Data points with different colors indicate different clinical strains tested. In general, EAEC/UPEC strains formed biofilms at modest levels, even though the strain 63.1 had formed biofilms at levels similar to those formed by prototypes strains when tested in urine.

In order to assess if hybrid genotypes improve the ability to form biofilms, the adherence to abiotic surface (in 96-well culture plates) of EAEC/UPEC strains was tested and compared to the adherence of 77 UPEC strains (Figure 2). In general, biofilm formation was higher in DMEM (median = 0.536 with third quartile [Q3] = 0.853) than in human urine (median = 0.391 with Q3 = 0.475) (*p* < 0.001—Kruskal-Wallis test). Hybrid EAEC/UPEC formed biofilms at modest, but perceptible levels either in DMEM (median = 0.535 with Q3 = 0.731) or in urine (median = 0.270 with Q3 = 0.411). Just six strains showed an invariable ability to form biofilm despite the tested conditions (DMEM or human urine), including the hybrid EAEC/UPEC strain 63.1 (mean of 0.535 ± 0.76 in DMEM and 0.553 ± 0.026 in urine) (Figure 2). When compared with the EAEC/UPEC strains 17.1 (genotype: *aatA aggR aapA fyuA chuA pap csgA ag43*) and 85.1 (genotype: *aatA aggR aapA fyuA chuA pap csgA*), the EAEC/UPEC strain 63.1 (genotype: *aatA aggR aggA aapA fyuA vat focA pap csgA ag43*) distinguishes from them for harboring the EAEC-specific fimbria AAF-I (*aggA*) and *focA*. In comparison to the prototype EAEC strains 17-2 (also positive for AAF/I) and 042 (positive for AAF/II), the strain 63.1 formed biofilms at similar levels when tested in human urine (Figure 2).

## Discussion

EAEC is a heterogeneous category that has been recognized for gathering versatile pathogens since the late 1990's, when their epidemiological association with diarrhea involving children, adults, HIV-infected patients and travelers became more and more evident (Kaper et al., 2004). Besides that, EAEC strains has been recently reported as a sporadic etiological agent in extraintestinal infections, mainly in urinary tract infections (UTI). In Brazil, a previous epidemiological study on UTI showed that 3.5% (8/225) of the strains were classified as EAEC based on the presence of the virulence marker *aatA* (Abe et al., 2008). Along with others classical UPEC markers, 5 of out 8 *aatA*-positive EAEC strains also carried UPEC gene *pap* (pyelonephritis-associated pilus) along with EAEC gene *aggA* (aggregative adherence fimbriae I – AAF/I), which highlighted the hybrid aspect of these strains (Abe et al., 2008). This epidemiological scenario is similar to the results found now in our study. In our collection, 3.5% (9/258) of the strains were positive for *aatA* as well as for *pap*. Moreover, three of these hybrid EAEC/UPEC strains harbored *aggA* (EAEC fimbria AAF-I) along with *pap* (UPEC pilus).

The gene *pic* (standing for protein-involved in colonization) was initially identified in the EAEC prototype strain 042 as a mucinase that displayed a pivotal role in the colonization of the intestinal mucus layer during diarrhea (Harrington et al., 2009). However, further experimental and epidemiological data broadened the role of *pic* endorsing its importance in extraintestinal infections such as UTIs, pyelonephritis and sepsis (Boll et al., 2013; Abreu et al., 2015). The gene *pic* was the most frequent EAEC marker detected among UPEC strains, reaching 13% of positivity in the Abe's study (Abe et al., 2008). Here, *pic*-positive strains accounted for 4.3% of the strains. Despite the low overall prevalence, here *pic* was exclusively detected in strains belonging to the extraintestinal phylogroup B2 and, in this group, *pic*-positive strains accounted for 11.5% of the isolates.

Studies on the distribution of plasmid and chromosomal genes of EAEC proposed that some EAEC strains belonging to lineages diverse from typical EAEC strains can acquire and maintain the pAA plasmid, the major virulence element in EAEC strains (Jenkins et al., 2005). Therefore, the Group 2 of EAEC, as defined by Jenkins et al., comprises a more heterogeneous group of EAEC strains that emerges from acquisition of pAA by strains that does not retain chromosomal markers of typical EAEC strains (Jenkins et al., 2005). In order to explain the emergence of hybrid EAEC/UPEC strains, some authors have fomented similar discussions. Here, we showed that different alternatives for the emergence of hybrid EAEC/UPEC strains isolated from UTI could exist. Based on the phylogenetic analyses carried out with concatenated chromosomal genes, we showed that two hybrid EAEC/UPEC strains share a chromosomal backbone of UPEC strains. Therefore, this finding suggest that mobile EAEC genetic markers posed on pAA plasmid (*aatA, aggR*, and *aapA*) can be acquired by UPEC strains. Concerning the EAEC/UPEC strains 17.1 and 85.1 (both classified as phylogroup D-ST3[ST69]), our findings conflict with the paradigm stablished in the literature that poses that diarrheagenic virulence markers are rarely detected in UPEC strains from phylogenetic group D-ST3[ST69] (Blanco et al., 2011; Olesen et al., 2012; Skjøt-Rasmussen et al., 2013). However, a Wallace-Gadsden's report endorses our findings, showing that the ST69 complex has a propensity to acquire EAEC virulence genes and so it may have evolved as a progenitor lineage from which UPEC and EAEC strains belonging to ST69 emerged (Wallace-Gadsden et al., 2007).

On the other hand, *E. coli* lineages that frequently gather commensal strains (phylogroups A and B1) may also include highly virulent UPEC strains. The hybrid EAEC/UPEC strain 63.1, characterized for having the most complete set of tested virulence genes for EAEC (*aatA, aggR, aapA*, and *aggA*) and UPEC (*fyuA, vat, focA*, and *pap*), besides being positive for *csgA* and *ag43*, was classified as phylogroup A. *E. coli* strains with similar genetic arrangements have already been associated with UTI outbreaks. In 1991, it was detected in Copenhagen an UTI outbreak involving a hybrid EAEC/UPEC strain (Olesen et al., 2012). Further molecular characterization showed that 18 out of the 19 outbreak strains were positive for the EAEC specific genes *aatA, aggR, aagA*, and *aap* along with the UPEC genes sat and *fyuA*. Additionally, phylogenetic analyses showed that the hybrid EAEC/UPEC outbreak strains belonged to intestinal phylogroup A, which is known to gather commensal strains (Olesen et al., 2012).

The formation of biofilms is considered to be a pivotal step during infection processes developed in human mucosas. EAEC strains are renowned for their prolific ability to form biofilms, which are in general supported by the aggregative adherence fimbriae (AAFs) coded on the plasmid pAA. Cases and outbreaks of UTI involving heteropathogenic EAEC/UPEC strains have endorsed the suspicion that EAEC virulence genes add uropathogenic properties to *E. coli* strains. Concerning EAEC/UPEC strains involved in the Copenhagen UTI outbreak (genotype: *aatA aggR* aap *aggA fyuA pic*; phylogroup A) (Olesen et al., 2012), it was shown that the expression of AAF/I (gene *aggA*) allowed a pronounced increase of bacterial adherence to human bladder epithelial cells as well as it allowed biofilm formation at levels significantly higher than those of UPEC prototype strains (Boll et al., 2013). In our study, the AAF/I-positive EAEC/UPEC strain 63.1 (genotype: *aatA aggR aggA aapA fyuA vat focA pap csgA ag43*; phylogroup A) showed an ability to produce invariable and perceptible biofilms in both DMEM and human urine. Although the biofilm levels had not been higher than those produced by the prototype EAEC strain 17-2 in DMEM, EAEC/UPEC strain 63.1 formed biofilms at higher levels than those formed by the strain 17-2 and at similar levels to those formed by prototype UPEC strains when tested with human urine.

Whether or not hybrid EAEC/UPEC strains have potential to cause diarrhea is an issue to be further evaluated. However, studies based on the extensive genetic characterization of typical EAEC strains have shown that phylogenetic patterns similar to those detected here are also found in diarrhea-associated EAEC strains. EAEC strains with phylogroup D and genotype *aatA aggR aapA fyuA chuA* were recovered from diarrhea and included the prototype strain 042 (Czeczulin et al., 1999; Okeke et al., 2010). Additionally, EAEC strains with phylogrup A and genotype *aatA aggR aapA fyuA* were also recovered from cases of diarrhea (Czeczulin et al., 1999; Okeke et al., 2010), including the strain C1192-92 recovered from a diarrhea case around the time of the UTI outbreak that occurred in F Compenhagen in 1991 (Olesen et al., 2012).

*E. coli* has a remarkable adaptation capacity that is mainly relied on its genomic plasticity (Touchon et al., 2009). Having a pan-genome with around 17,000 genes and 4700 genes per genome in average, no single *E. coli* strain can be assumed as a representative of the species. In this scenario, applying the genetic boundaries stablished for archetypal pathotypes in epidemiological studies involving *E. coli* has shown to be an elusive goal. Events involving hybrid or heteropathogenic *E. coli* strains have endorsed this perspective and blurred the classification of *E. coli* strains into classical pathotypes.

## Author contributions

AP conceived the study and designed the experiments. AP and FL wrote the manuscript and were responsible for concepts, vision and direction of the study. FL, DN, Pd, and MA performed geno- and phylotyping experiments and analyzed the data. AP, Cd, and DN carried out the genetic sequencing. FC and Ld performed biofilm assays. LF and FL carried out the isolation and identification of *E. coli* strains. All authors read and approved the final manuscript.

## Funding

This work was supported by the Fundação de Apoio à Pesquisa do Distrito Federal (FAP-DF) with the grants No 193.000.019/2012 and 193.001.042/2015.

### Conflict of interest statement

The authors declare that the research was conducted in the absence of any commercial or financial relationships that could be construed as a potential conflict of interest.

## Abbreviations

AEAC: enteroaggregative *E. coli*
UPEC: uropathogenic *E. coli*
ExPEC: extraintestinal pathogenic *E. coli*
UTI: urinary tract infection
ST: sequence type
VG: virulence gene.
