# Supplementary material for: Virulence Markers and Phylogenetic Analysis of Escherichia coli Strains with Hybrid EAEC/UPEC Genotypes Recovered from Sporadic Cases of Extraintestinal Infections
Source: Front Microbiol. 2017 Feb 3;8:146. doi: 10.3389/fmicb.2017.00146 (PMC5290387; doi:10.3389/fmicb.2017.00146)
Supplement: Supplementary file 1 [file Table1.docx]

**Supplementary Table 1. Genotypes of clinical ExPEC strains tested in biofilm assays**

| **Genotypes** | | **Phylogenetic groups (N)** |
| --- | --- | --- |
|  | *chuA csgA* | D (2); B2 (1); ND* (1) |
|  | *csgA* | B1 (3); A (2) |
|  | *focA* | B1 (1) |
|  | *fyuA chuA csgA* | B2 (5); D (5) |
|  | *fyuA chuA focA pap csgA* | D (2) |
|  | *fyuA chuA pap* | B2 (1) |
|  | *fyuA chuA pap csgA* | D (6) |
|  | *fyuA chuA pap csgA ag43 pCVD aggR* | D (1) |
|  | *fyuA chuA pap csgA ag43 pilS* | D (2) |
|  | *fyuA chuA pap csgA pCVD aggR* | D (1) |
|  | *fyuA chuA vat csgA* | D (1) |
|  | *fyuA chuA vat pap csgA ag43* | D (1) |
|  | *fyuA chuA vat pap sfa cnf csgA* | B2 (1) |
|  | *fyuA csgA ag43* | B1 (1) |
|  | *fyuA vat csgA* | A (1) |
|  | *fyuA vat focA csgA* | A (1) |
|  | *fyuA vat focA pap csgA ag43 pCVD aggR* | A (1) |
|  | *fyuA yfcV chuA* | B2 (1) |
|  | *fyuA yfcV chuA csgA* | B2 (2); D (2) |
|  | *fyuA yfcV chuA pap csgA* | D (7) |
|  | *fyuA yfcV chuA vat focA pap csgA ag43 pilS* | B2 (1) |
|  | *fyuA yfcV chuA vat focA pap sfa cnf csgA* | B2 (1) |
|  | *fyuA yfcV chuA vat focA pap sfa cnf csgA ag43 pilS* | B2 (1) |
|  | *fyuA yfcV chuA vat focA pap sfa cnf csgA pic pilS* | B2 (1) |
|  | *fyuA yfcV chuA vat focA pap sfa cnf pic* | B2 (1) |
|  | *fyuA yfcV chuA vat focA sfa csgA pic* | B2 (1) |
|  | *fyuA yfcV chuA vat pap cnf csgA* | B2 (1) |
|  | *fyuA yfcV chuA vat pap cnf csgA ag43 pilS* | B2 (1) |
|  | *fyuA yfcV chuA vat pap cnf csgA pic* | B2 (1) |
|  | *fyuA yfcV chuA vat pap csgA* | B2 (5) |
|  | *fyuA yfcV chuA vat pap sfa cnf csgA* | B2 (2) |
|  | *fyuA yfcV chuA vat sfa cnf csgA pic* | B2 (1) |
|  | *fyuA yfcV pap csgA ag43* | B1 (1) |
|  | *fyuA yfcV vat csgA* | A (1) |
|  | *pap csgA* | B1 (1); A (1) |
|  | *vat csgA* | B1 (1) |
|  | *yfcV chuA vat csgA* | D (1) |
|  | *yfcV chuA vat pap csgA* | B2 (1) |
|  | *yfcV vat focA csgA* | A (1) |
|  | Total | 77 |

* Not determined
